# Temporal variation may have diverse impacts on range limits

**DOI:** 10.1098/rstb.2021.0016

**Published:** 2022-04-11

**Authors:** Robert D. Holt, Michael Barfield, James H. Peniston

**Affiliations:** Department of Biology, The University of Florida, Gainesville, FL 32611, USA

**Keywords:** temporal variability, Allee effect, range limit, source–sink, gradient, Jensen's inequality

## Abstract

Environmental fluctuations are pervasive in nature, but the influence of non-directional temporal variation on range limits has received scant attention. We synthesize insights from the literature and use simple models to make conceptual points about the potentially wide range of ecological and evolutionary effects of temporal variation on range limits. Because organisms respond nonlinearly to environmental conditions, temporal variation can directionally alter long-term growth rates, either to shrink or to expand ranges. We illustrate this diversity of outcomes with a model of competition along a mortality gradient. Temporal variation can permit transitions between alternative states, potentially facilitating range expansion. We show this for variation in dispersal, using simple source–sink population models (with strong Allee effects, or with gene flow hampering local adaptation). Temporal variation enhances extinction risk owing to demographic stochasticity, rare events, and loss of genetic variation, all tending to shrink ranges. However, specific adaptations to exploit variation (including dispersal) may permit larger ranges than in similar but constant environments. Grappling with temporal variation is essential both to understand eco-evolutionary dynamics at range limits and to guide conservation and management strategies.

This article is part of the theme issue ‘Species’ ranges in the face of changing environments (Part II)’.

## 1. Introduction

All species have spatially restricted ranges [1]. Elucidating what causes range limits is an issue at the interface of biogeography, ecology and evolutionary biology. Principal explanations for range limits include constraints on dispersal and spatial heterogeneity in abiotic and biotic factors governing population persistence. That is to say, range limits arise in part because species' niche requirements (which when met allow births to exceed deaths at some densities, [2]) are not satisfied everywhere. A huge literature exists exploring these two explanations and weighing their relative importance (e.g. [3–5]). Species’ niche requirements can evolve, as can dispersal propensities, leading to range shifts reflecting evolutionary processes [6]. Conversely, if a species' range limits are stable over long time scales, its niche is probably evolutionarily conservative [7].

Another broad generalization is that demographic rates governing ranges (births, deaths and dispersal) vary through time, driven by changes in abiotic conditions (e.g. drought [8], deluges [9]) or biotic factors (e.g. variation in shark numbers produces fluctuating predation on prey [10]). Organisms have evolved myriad traits for handling variation [11], but extreme events [12] can push them beyond their adaptive limits. One general insight from existing theory on temporal variation in ecology and evolution is that variation is not just statistical ‘noise’ but has important qualitative effects (e.g. on species coexistence). Here, we synthesize previous studies and present illustrative model results demonstrating the same is probably true for range limits. We suggest temporal variation matters for range limits for several reasons. One is the pervasive nonlinearity of organismal responses to environmental conditions and population density (making long-term growth rates sensitive to variance). Fluctuations also amplify the impacts of demographic stochasticity, extreme events, and metapopulation processes. Moreover, variation can influence evolutionary processes, such as the interplay of gene flow and selection in determining local adaptation.

We focus on aspects of variation not involving long-term secular trends (e.g. directional climate change), or singular abrupt events. The examples we explore include periodic and deterministic variation (e.g. seasonality), or stochastic variation with different degrees of predictability around a long-term mean (e.g. red noise [13]). An important task for future work will be to compare consequences for range limits of different patterns of temporal variation; here we emphasize insights that are robust to changes in the exact character of the variation.

A considerable literature examines how temporal variation influences population dynamics and evolution, but little attention has been explicitly focused on how temporal variation might impact range limits. We draw upon this literature to make conceptual points and use simple models that illustrate the potentially diverse impacts of temporal variation on range limits. Simple models provide essential conceptual anchors in the complex sea of ecological and evolutionary processes. They help crystallize understanding, highlighting phenomena warranting closer attention in more complex, realistic models. There are many precedents. One can point to how May [14] used the simple discrete-time logistic model to illustrate how time-lagged density dependence generates chaotic dynamics—an enduring message about unpredictability in ecological systems which may help explain hyperdiversity [15]. In spatial ecology, Skellam's simple model [16] for exponentially growing populations with diffusive movement across one-dimensional, homogeneous habitats provided foundational insights informing a large body of theoretical and empirical literature on invasions [17]. The simple source–sink model of Gomulkiewicz *et al*. [18] showed that in a sexual species with a major gene locus, modest immigration facilitates adaptive evolution and persistence in a sink by infusion of genetic variation, but higher immigration could degrade local adaptation, given negative density-dependence. These suggestions were later verified using more complex quantitative genetic models of evolution along gradients [19].

In this spirit, we use simple models to explore how temporal variation influences range limits. We present illustrative results for species distributed along one-dimensional continuous gradients [20,21], and in discrete habitats coupled by dispersal (source–sink models, [22]). Even gradual gradients can exhibit abrupt transitions in vegetation (e.g. cloud forest boundaries, [23]), with many species having range limits concordant with such habitat transitions. Discrete habitat models approximate dynamical processes across such boundaries. Understanding simple source–sink models has proved useful in empirical studies of invasions and range limits, particularly for species inhabiting inherently patchy environments (e.g. [24]). We suggest messages from such models inform understanding of the eco-evolutionary dynamics of range limits, more broadly.

## 2. Temporal variation and range limits: ecological considerations

Evolutionary dynamics play out on templets defined by a species' basic ecology, and considering ecological issues provides essential context for understanding eco-evolutionary dynamics. We start by considering how temporal variation influences distributional limits for ecological reasons, without evolution. Consider a thought experiment. For simplicity, assume dispersal into each location is a trickle, sufficing to ‘seed’ localities with a species over historical time scales, but not quantitatively important in local dynamics thereafter. A species' range is that set of those locations where it can persist, without immigration, following initial colonization. Along a temporally constant gradient, one end extends beyond the niche. Add temporal variation. Will the range shrink, or expand?

Holt *et al*. [21] proposed three classes of demographic reasons for range limits (i) deterministic niche limits, (ii) demographic stochasticity at very low carrying capacities, and (iii) extreme events causing extinction. We revisit these reasons for range limits, exploring the implications of incorporating temporal variability.

### (a) Range limits occur because environments are outside a species’ realized niche

A range limit can arise because a species' niche requirements are not met: i.e. its intrinsic growth rate becomes negative along a gradient [4]. Local growth rates depend upon local abiotic conditions, resources, predators and so forth, entering population growth models as parameters or variables. For species *i* in location *x*, assuming continuously overlapping generations, its growth rate is:

2.1 $$\frac{1}{N_{i}(t,x)}\frac{dN_{i}(t,x)}{dt}=b_{i}(t,x)-d_{i}(t,x)=r_{i}(t,x).$$

Here, *b* and *d* denote instantaneous birth and death rates. In a temporally constant environment, and without Allee effects ([2] and see below) the species’ range is those locations where, when rare, its intrinsic growth rate is

2.2 $$r_{i}(x)>0.$$

This invasibility criterion is at the heart of analyses of assembly processes and coexistence in community ecology [25]. Range limits occur where expression (2.2) changes sign across space.

In a variable environment, average growth rate over a long interval *T* is

2.3 $$\bar{r_{i}}(x)=\frac{1}{T}\int_{0}^{T}r_{i}(t,x)\;dt.$$

As a frame of reference, we compare ranges expected for variable environments to constant environment with time-varying parameters set at their time-averaged means. The range is comprised of locations where

2.4 $$\bar{r_{i}}(x)>0.$$

If differing from those demarcated by (2.2), temporal variation impacts range limits. Using time-averaging [26] and applying Jensen's inequality [27] permits qualitative assessment of how temporal variation alters long-term average growth rates (and thus range limits), compared to constant environments. Jensen's inequality states that the impact of variation depends upon growth rate curvature, as a function of a quantity. If the growth rate function is concave up and the quantity has mean *μ*, time-averaged growth rate exceeds the function evaluated at *μ*. For a concave down function, the average is below the growth rate at *μ*.

This approach pertains to any model of local interactions. As a concrete example, we revisit a competition model explored in [28]. Two consumer species with densities *N_i_*(*x*, *t*) (*i* = 1,2) compete for an abiotic resource of abundance *R*(*x, t*) along a gradient *x* in density-independent mortality *M*(*x*) (e.g. imposed by generalist predation). Each consumer has a saturating functional response. In a constant environment, one consumer (species 2) has higher attack rate on the resource, the other (species 1) a lower handling time. The model is:

2.5 $$\begin{array}{rl} \frac{dN_{i}(x,t)}{dt} & =N_{i}(x,t)\\ & \;\times \left(\frac{b_{i}a_{i}(t)R(x,t)}{1+a_{i}(t)h_{i}(t)R(x,t)}-m_{i}-M(x,t)\right) \end{array}$$

and

2.6 $$\begin{array}{rl} \frac{dR(x,t)}{dt} & =I-dR(x,t)\\ & \;-\sum_{i}^{2}\frac{a_{i}(t)R(x,t)}{1+a_{i}(t)h_{i}(t)R(x,t)}N_{i}(x,t). \end{array}$$

**Figure 1.**
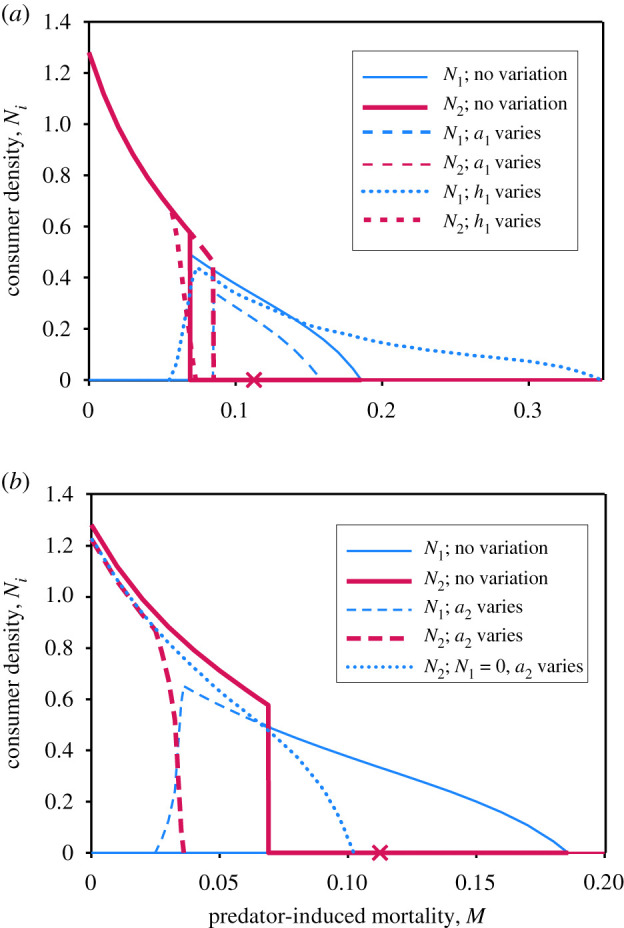
Competitor abundances along a density-independent mortality gradient (*M*). (*a*) Constant attack rates and handling times (solid curves) contrasted with *a*_1_ varying, while *a*_2_ and *h*_1_ are constant (dashed curves); or, *h*_1_ varying while *a*_1_ and *a*_2_ are constant (dotted curves). (*b*) As (*a*), except dashed curves have *a*_2_ varying with constant *a*_1_ (constant handling times). Species coexist between *M* = 0.025 and 0.035. Dotted curve shows *N*_2_ when species 2 is alone and *a*_2_ varies. Bold × indicates maximal *M* allowing species 2 persistence, were it alone and parameters constant. *I* = 0.1 and *d* = 0.1. Intrinsic mortalities are *m*_1_ = 0.1 and *m*_2_ = 0.075. When attack rate is constant, *a*_1_ = 2, *a*_2_ = 3. For variable attacks, *a*_1_ = 2[1 + *b* sin(2π*ft*)] or *a*_2_ = 3[1 − *b*sin(2π*ft*)], where *b* = 0.9 and *f* = 0.02. Handling times are *h*_1_ = 3 [or 3[1 + *b*sin(2π*ft*)] for dotted curves in panel (*a*)] and *h*_2_ = 5. Blue lines show species 1 and red lines show species 2 density.

Here, *a_i_*(*t*) is attack rate, *h_i_*(*t*) handling time, *b_i_* converts consumption to births and *m_i_* is intrinsic mortality (total mortality is *m_i_* + *M*(*x, t*)). In a constant environment, the model predicts local competitive exclusion, and parapatric distributions along a mortality gradient [28] (figure 1, solid curves). Species 2 dominates at low mortality, with abrupt transition to species 1 at higher mortality. The ‘ × ’ in the figure shows the range margin of species 2, when alone.

We now introduce deterministic, periodic variation. Growth rate varies linearly with density-independent mortality (zero curvature), so using Jensen's inequality, temporal variation in mortality factors does not alter range limits for each species when alone (this conclusion neglects demographic stochasticity; see below). Variation in other parameters *does* influence range limits. Consider species 1 alone. The right-hand side of figure 1*a* shows representative examples. Birth rates as a function of attacks are concave downwards, so time-averaged births are lessened. Species 1 thus shrinks its range at the high mortality end of the gradient (thin dashed line, compared to thin continuous line). The opposite occurs with temporal variation in handling time. Growth rate as a function of *h_i_* is concave upwards, so variation boosts time-averaged growth rate, permitting a larger range (right-hand dotted tail in figure 1*a*). In Richard Levins' [26] memorable phrase, this species ‘eats variance’, allowing populations to persist in this part of the gradient, which would go extinct in the absence of such temporal variance.

Adding a competitor produces indirect effects of temporal variation on range limits. Figure 1*a* shows that variation in attacks in species 1 reduces its range, and increases that of its competitor. Conversely, variation in handling time in species 1 expands its range and shrinks that of species 2 (with a zone of coexistence). In figure 1*b*, species 2 experiences variable attacks. Were it alone, it would experience modest range decline (dotted line, compared to ‘x’), but presence of a competitor generates a larger range contraction (dashed lines). So, interspecific interactions can modulate how temporal variation influences ranges. A more comprehensive analysis would include mechanistic submodels relating environmental drivers (e.g. temperature) to parameters (e.g. attack rates), examine covariation across a broad parameter space (in both species), contrast different patterns of temporal variation and explore alternative community states (which can occur when species' attack rates vary out-of-phase; details not shown). Here, we use this familiar exploitative competition model simply to illustrate how temporal variation can leave unchanged, shrink, or even expand species’ ranges along gradients.

In a low-dispersal limit (so there are no sink populations or metapopulation dynamics, as assumed in figure 1), local communities contain only species that can coexist locally. Community ecologists have demonstrated that fluctuating environments may permit coexistence, whereas in constant environments, exclusion occurs [29,30]. Elucidating how nonlinear responses to resources, internal storage [31] and life-history buffering (allowing temporal partitioning of niches) mediate competitive interactions in variable environments is a major objective of coexistence theory [25]. Eliminating temporal variation in such systems entails local extinctions, altering range limits. Sometimes, however, temporal variation disrupts coexistence. Noonburg & Abrams [32] demonstrated that resource pulses destabilize systems where coexistence involves keystone predation. Species coexisting in a constant environment may thus not co-occur in similar but variable environments. The interplay of interspecific interactions and temporal variability is likely to have consequences for range limits, differing among taxa and locations, in some situations expanding them, in others, shrinking them. This is a significant challenge for future work.

### (b) Range limits can reflect strong density dependence and demographic stochasticity

The above arguments do not consider finite population sizes and the stochastic vicissitudes of individual births and deaths. Along a gradient in carrying capacity *K*, where *K* gets small because of strong, negative density dependence, range limits could arise from extinction due to demographic stochasticity [21]. With density-independent growth up to *K*(*x*), above which growth stops in a constant environment, mean time to extinction *T*_e_ scales with *K*(*x*) as *T*_e_ = *c*e*^aK^*^(*x*)^ [33]. Populations with very small *K* have short expected lifespans; recurrent colonization is needed for such locations to be within a species' realized range. The exponential dependency, however, implies that even modestly abundant populations persist for long periods in constant environments. Temporally varying *K* dramatically magnifies effects of carrying capacity on extinction due to demographic stochasticity; a large average *K* may still go along with high extinction risk [27]. Locations with large magnitude variation in carrying capacities will thus probably be lost from species’ ranges.

### (c) Range limits can reflect transient extinction events

Fluctuations in birth or death rates can push even large populations to episodes of low abundance, where demographic stochasticity causes extinction. One aspect of temporal variation may be a greater incidence of extreme events. Holt *et al*. [21] briefly note that range limits could arise from episodic extreme events (e.g. droughts, windstorms) causing pulses of very high mortality or low births, and so rapid extinction. Empirical studies increasingly demonstrate the importance of extreme events in determining range limits (e.g. [34]). Standard metrics of variability such as variance may matter less than the statistics of extreme values in governing range limits.

### (d) Metapopulation effects

We have so far focused on largely closed populations. Locations can experience extinctions yet remain within the range, given recurrent colonization in a metapopulation [35,36]. The interplay of dispersal and local temporal variability can foster species persistence (and coexistence), impossible in otherwise similar but temporally invariant environments. Temporal variation in growth rate in sinks maintained by immigration (where average growth rate is negative) increases time-averaged abundance, particularly given positively autocorrelated variation [37]. This creates runs of bad years, when immigration sustains the sink and runs of good years when exponential growth permits it to surge. Numerically, the latter dominates in determining average abundance. Roy *et al*. [37] showed theoretically (and [38] experimentally) that given intermediate levels of dispersal this ‘inflationary effect’ permits metapopulation persistence, despite each local population being on average a sink (so extinction is inevitable in a constant environment). Along a gradient of increasingly severe environments, range limits reflecting metapopulation dynamics may extend further, given temporal variability.

### (e) Dispersal variability

Temporal variation in dispersal (which is ubiquitous) can alter range limits in a variety of ways. For instance, many near-shore marine organisms, sedentary as adults, have pelagic larvae or gametes. Though larvae can influence dispersal kernels through swimming behaviours, ocean currents significantly affect dispersal direction and distance [39]. The great dynamism in currents (e.g. mesoscale eddies and directional jets varying across many spatio-temporal scales [40]) generates substantial variability in the distance and even directionality of dispersal. Gaylord & Gaines [40] demonstrated offshore flows could create coastal range limits by imposing emigration losses on dispersing larvae. They suggested fluctuating flows could change impenetrable barriers to leaky ones, permitting range expansions. Jin *et al*. [41] developed a model for species inhabiting rivers with alternating pools and riffles. The average flow regime is great enough that in a homogeneous river, a species cannot invade upstream. With seasonal flow fluctuations, around this average, an ‘invasion ratchet’ can occur, where a species moves through riffles at times of low flow, and persists in pools during high flows, permitting episodic expansion upriver. Temporal variation in marine metapopulations sometimes reduces metapopulation growth [42], but temporal alternation between distinct dispersal regimes—each that separately predicts extinction in a constant environment—can also permit metapopulation persistence in fluctuating environments [43]. Geographical ranges may expand, given particular patterns of variation in dispersal and connectivity.

**Figure 2.**
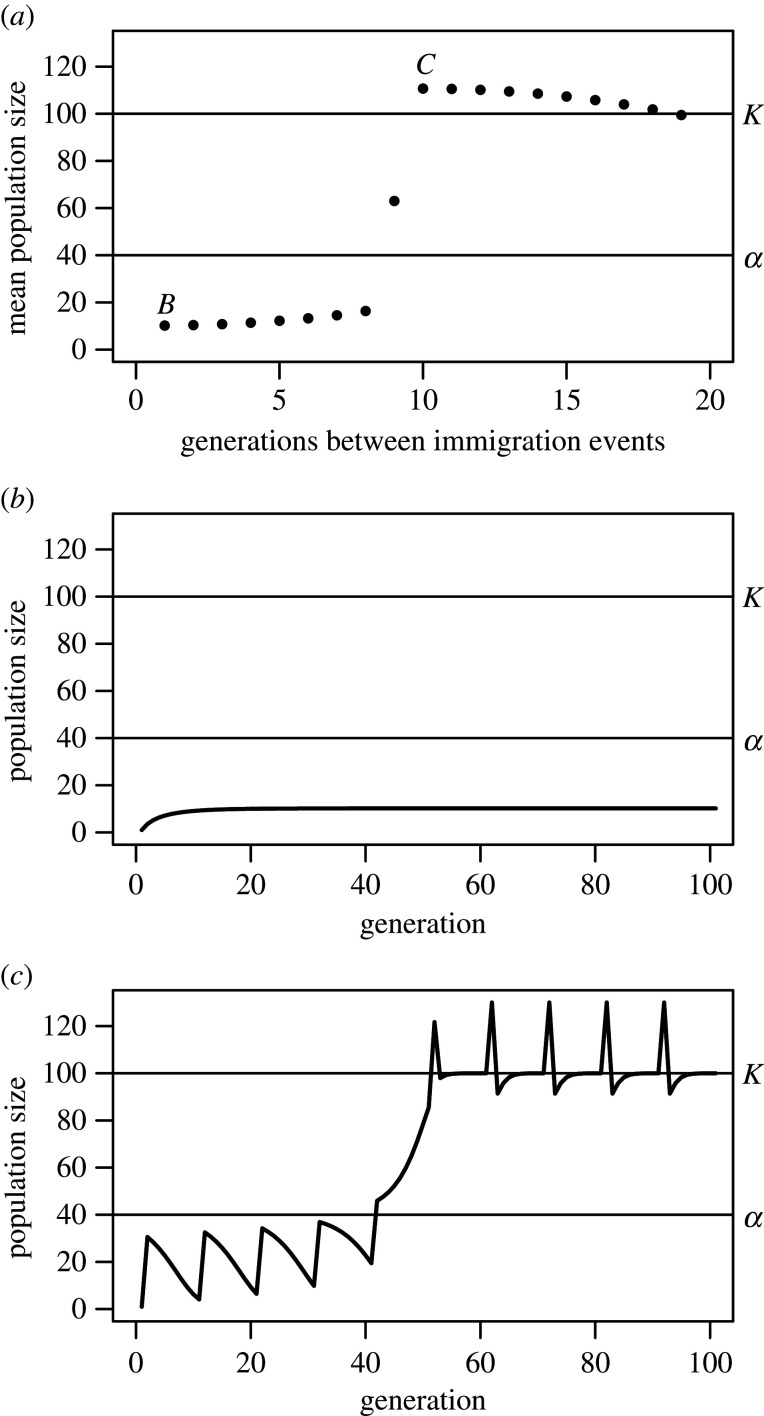
Temporal spacing of immigration influences establishment in a sink with an Allee effect. Cumulative immigrants per run of 100 generations was 300; initial density was 1. Immigration pulses were larger in runs with fewer pulses. (*a*) Effect of immigration spacing on mean abundance, averaged over the last immigration cycle. (*b*) and (*c*) show typical trajectories. (*b*) Constant immigration (three per generation)—the population stays trapped at low numbers (were immigration eliminated, extinction ensues). (*c*) Pulses each 10 generations; the population ratchets towards establishment; i.e., persistence without immigration. Intrinsic growth rate *r* = 1.1, *α* (Allee threshold) = 40, carrying capacity *K* = 100. See the electronic supplementary material for details and explorations of parameter space.

Temporal variation in dispersal can facilitate range expansion if there is strong positive density dependence at low numbers—Allee effects [44,45]. Keitt *et al*. [45] showed Allee effects could produce stable range limits in patchy landscapes, even if all patches are intrinsically equal. They suggested that temporal variation in dispersal might foster expansion. We illustrate this suggestion with a discrete generation model including Allee effects and immigration (details in the electronic supplementary material, S1). The species initially occupies some patches within a landscape, at carrying capacity; these patches provide a unidirectional immigrant flow into a focal marginal patch (a sink where low immigration initially maintains abundance below an Allee threshold). We introduce temporal variation in immigration into this ‘black hole’ sink, assuming periodic pulses, with cumulative immigrant numbers held constant (so the mean number of immigrants per unit time is constant). Immigration episodes are either frequent, small immigration events (low or no variation), or less frequent but larger (higher variation); the latter allows populations to grow and persist (figure 2*a*). With small pulses each generation, the sink population remains below its Allee threshold and never establishes (figure 2*b*). With infrequent, larger pulses, the population overcomes that threshold and grows (figure 2*c*), permitting persistence were immigration stopped. A single large pulse might circumvent the threshold in one fell swoop. In figure 2*c*, immigration pulses induce an invasion ratchet. The initial pulse does not surmount the threshold, but the rate of decline lessens at higher density. At the next pulse, the population starts higher, and is again boosted, further shrinking its rate of decline. Finally, the population circumvents its Allee threshold and establishes. Note that these effects would not occur if immigration events of the same size were simply temporally spaced out (which would decrease the total number of immigrants, and the average number of immigrants per unit time) because establishment requires that immigration pulses (singly or consecutively) are large enough to overcome the Allee threshold. As discussed in the electronic supplementary material, S1, because of time-lagged density dependence in the model, large pulses can cause cycles and even risk extinction; moderate variability in immigration is optimal for establishment. The interactive effects of density dependence and temporal variation in dispersal on range limits should be examined for a wider range of models and more complex spatio-temporal scenarios [46].

## 3. Implications of temporal variation for range limits: evolutionary considerations

Traits determining how a species responds to temporal variability emerge from the interplay of microevolutionary processes such as selection, drift and gene flow. Range limits may arise because genetic variation needed for persistence beyond range margins is simply absent [47], or because drift depletes variation [48], or because gene flow hampers selection [49]. Temporal variation can influence all these processes. Evolution can influence ecological mechanisms that lead to range limits, leading to changes in those range limits. For instance, natural selection often (not always [50]) increases local population size; if so, local populations become less vulnerable to extinction. This provides one indirect route through which natural selection can influence range limits.

### (a) Genetic variation

Consider local populations with scant immigration. The amount of genetic variation available for selection depends upon effective population size. Most natural populations have effective sizes below their census sizes [51]; temporal variation in abundance shrinks effective size [52]. Classic work [53] showed harmonic mean abundance governs the rate of loss of heterozygosity in fluctuating populations. The harmonic mean is dominated by low numbers, suggesting temporal variation in abundance may hamper range expansion by loss of genetic variation permitting local adaptation. Moreover, selection weakens against drift, corroding adaptation in marginal environments. In metapopulations, declines in local genetic variation can be quickly replenished if migration rates from other patches is high; however, if an environmental change occurs synchronously over the entire metapopulation, local genetic variation may never be replenished [54]. Therefore, in metapopulations the effects of fluctuations on genetic variation (and local adaptation) will probably depend on the migration rate and the spatial autocorrelation of variation [54]. Interestingly, positively autocorrelated variation in local growth rates can increase time-averaged abundance in sinks [37,38], which can boost cumulative births. If local adaptation depends upon novel mutations, there is greater scope for such variation arising in temporally varying, compared to constant, sink environments (as shown using formal birth-death models [55]).

### (b) Gene flow versus local selection: impacts of temporal variation

Nagylaki [56,57] examined single-locus selection in an island population with discrete generations receiving immigrants from an external source, with fluctuations in the rate of migration *Φ* (the fraction of a population consisting of immigrants). Nagylaki concluded that temporal variation in migration hampers retention of locally adapted alleles [57]. A simple example illustrates the basic idea. Assume in a constant environment that the fraction of a population comprised of immigration, after dispersal but before reproduction and selection, is *Φ* = 0.5. A locally adapted allele with a large selective coefficient can be retained. In a comparable environment with stochastic migration, with *Φ* = 0 half the time, and *Φ* = 1 half the time, the average migration rate is unchanged. But in any generation with *Φ* = 1, the prior generation is entirely expunged, and the population consists entirely of immigrants. Thus, alleles with high local selective advantages can be lost. Complementing this genetic argument, ecological forces can favour immigrants. Long *et al*. [58] argued (and demonstrated experimentally) that given clonal genetic variation, superior clones could be excluded given variation in local growth, because average abundance of an inferior competitor sustained by immigration is boosted by the inflationary effect [35]. Temporal variation thus fostered maladaptation.

Nagylaki's models are not explicit about the relationship of selection and population dynamics. Models explicitly including population dynamics suggest that sometimes, temporal variation helps adaptation to a sink environment, facilitating range expansion. Holt *et al*. [59] used quantitative genetic models to examine how temporal variation in the optimal phenotype in a sink influenced adaptation and persistence there. Here we summarize their results. After reproduction, viability selection on offspring leaves *N_t_* surviving offspring, followed by immigration of *I* individuals from an external source. Random mating generates the next generation. The rate of gene flow, Φ = *I*/(*I* + *N*), is not a fixed parameter, because population size is dynamic, dependent in part upon selection. Alternative equilibria can arise because of this nonlinear relationship between population size and the force of gene flow in constant sink environments—one maladapted at low abundance, the other well-adapted at high abundance. In a harsh sink, where immigrants have low fitness, few individuals survive selection; gene flow is high, because *N* is low. In a mild sink, more individuals survive selection, and *N* will be higher, reducing gene flow. If viability selection is mild in one generation, *N* is larger the following generation. Adaptation increases *N*, reducing the ‘swamping’ effect of gene flow, allowing further adaptation and increases in *N*. Because of this positive feedback, moderate amounts of temporal variation in the sink can move a population from a maladapted to an adapted state, after which it can persist without immigration. This result emerged for variation in the selective optimum or in non-selective fitness components such as fecundity, for both periodic and stochastic variation [59]. The electronic supplementary material provides an example of moderate stochastic variation facilitating adaptation in a sink.

So sometimes, temporal variation in the degree of local maladaptation facilitates adaptive evolution, permitting a persistent high-density population, where in a constant environment, a sink population remains maladapted at low density. However, Holt *et al*. [59] also showed that large-magnitude variation could overwhelm this effect, even driving previously adapted populations to extinction. Overall, moderate amounts of temporal variation provided the greatest scope for adaptive evolution in a sink. We surmise this ‘Goldilocks’ effect often describes how temporal variation influences range limits; a moderate amount may extend the range, but large amounts probably shrink it.

**Figure 3.**
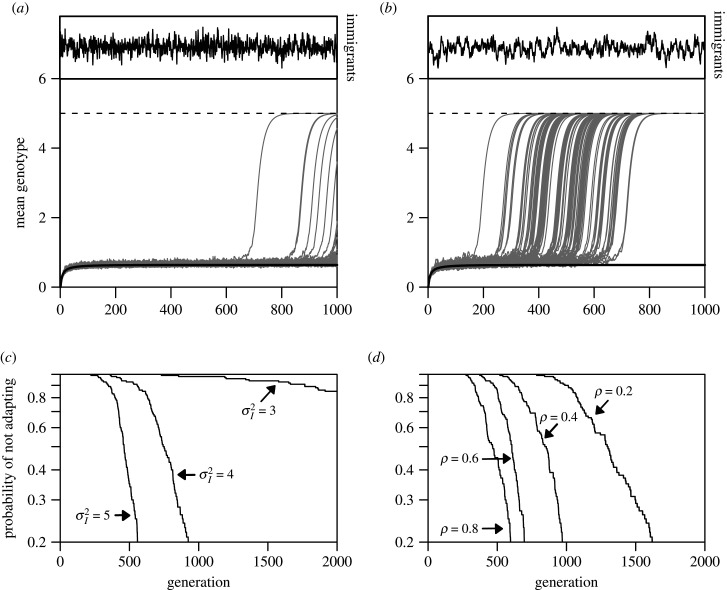
Effect of temporally varying immigration on adaptation to a sink, colonized at time 0. (*a*) and (*b*) illustrate evolutionary trajectories in sinks with temporally varying immigration ($\sigma_{I}^{2}=5.0$). Populations are initially maladapted, with immigrants having mean genotypic values of 0; the solid line shows the maladapted equilibrium, maintained by gene flow hampering selection. The dashed line is the local adapted equilibrium. In a constant environment the population stays maladapted. The ‘probability of not adapting’ by generation *t* is the fraction of runs still at or near the maladapted equilibrium*.* Serial autocorrelation of local optima (see the electronic supplementary material, S3 for details and other parameters) is *ρ* = 0.2 in (*a*), 0.8 in (*b*). Insets illustrate how immigration rates vary. (*c*) Influence of variation in immigration$\left(\sigma_{I}^{2}\right)$. (*d*) Effect of autocorrelated immigration (*ρ*).

Peniston *et al*. [60] recently evaluated how temporal variation in dispersal influences adaptation and persistence in a sink. They showed that pulsed (rather than continuous) movement from source to sink (for the given mean immigration rate) can facilitate adaptation. This occurs because immigration gaps provide periods when selection acts unimpeded by gene flow, increasing population size. This weakens the impact of gene flow on local adaptation during the next bout of immigration. These results match general theory [61] that temporal variation in dispersal rates fosters local adaptation. They [60] considered a specific form of variation in dispersal—regular pulses (as in the above Allee model). While this assumption matches some systems (e.g. organisms dispersed by episodic winter storms), in others, immigration fluctuates more continuously. We modified the model to investigate continuous variation in immigration. The details are in the electronic supplementary material, S3, but key features are as follows. In this discrete-generation model, fitness is governed by a quantitative trait. Variability in immigration is an autocorrelated Gaussian random sequence (*σ_I_*^2^ and *ρ* are variance and autocorrelation in immigration rates). Densities are assumed low enough to neglect density dependence. In a constant environment, the sink stays persistently maladapted. Figure 3*a* and *b* show typical evolutionary trajectories during which populations' mean genotypes move from a maladapted state to an adapted state in the sink, given variability. Increased magnitude of variation in immigration rates facilitates adaptation to sink environments, fostering range expansion (figure 3*c*), as does higher autocorrelation (figure 3*d*). This makes intuitive sense; highly autocorrelated immigration surges allow sink population growth during phases of high immigration, followed by phases of reduced gene flow, when selection acts largely unimpeded. In short, whether or not temporal variation in immigration into a sink is periodic or stochastic, highly autocorrelated or not, it can foster adaptation in a sink; differences in the character of variation matter quantitatively (compare figure 3*a* and *b*), but not qualitatively.

Not all variation in dispersal facilitates range expansion. As noted above, negative density dependence constrains population size; a large influx of immigrants lowers resident fitness, increasing the inhibitory impact of gene flow [18,60]. Highly variable immigration might even cause ‘migrational meltdown’ [62] with loss of adaptation and increased extinction risks at range margins. Moderate variation in dispersal, we surmise, is most likely to foster local adaptation and range expansion.

### (c) Beyond source–sink models and stable ranges

Much of the work we have touched on has focused on source–sink models, or range limits that have settled into some kind of equilibrium. This is likely because these models are relevantly simple to work with and have a long history of being used to understand range limits. However, these models are limited in their scope and valuable insights can be gained by modelling other landscapes and/or considering transient dynamics of ranges. For instance, Ellner & Schreiber [63] showed that temporal variation in dispersal could speed up invasions and that this could be amplified by certain patterns of temporal variation in birth/death rates.

Models of continuous landscapes and metapopulations have shown that dispersal evolution can play a significant role in range limits (e.g. Phillips & Perkins [64]). Kubisch & Poethke [65] used an individual-based model of a metapopulation to investigate how temporal variation influenced range sizes via evolution of dispersal rate, finding ranges are largest at moderate degrees of temporal variation; if variation becomes too large, ranges contract. Schreiber [66] showed ranges were greater at intermediate levels of temporal variation in a model where habitat selection strategies could evolve. One evolutionary driver for dispersal is spatio-temporal variation in fitness [67]. Dispersal barriers may more likely be breached when source populations experience variability in local conditions favouring higher local dispersal. Oldfather *et al.* [68] found empirical evidence that plants have greater dispersal at range margins when there is more temporal variability in plant demography. Sometimes, however, temporal variation favours lower dispersal [69]. Regardless of direction, a key indirect eco-evolutionary route through which temporal variation affects range limits is via dispersal evolution.

There is a large literature on eco-evolutionary dynamics along environmental gradients [70]. A classic result is that if the environment changes too quickly relative to the genetic variation available, range margins arise [49]. This occurs because with steep environment gradients local fitness, and thus density, rapidly declines near the range margins, depleting genetic variation by drift and making selection less effective [48]. Therefore, most migrants into peripheral populations might mainly come from the core of the species range where density is the highest. These migrants from the core are probably poorly adapted to the conditions at the range margin and unable to establish viable populations on their own, and can thus be a source of maladaptive gene flow hindering local population growth and adaptation, and thus creating range limits. Benning *et al*. argue that temporal variation in selective optima along continuous gradients magnify these prcoesses, and can generate stable range limits [71]. Insights from source–sink models, however, discussed above suggest that increasing the magnitude of either type of variation at least moderately might sometimes allow larger ranges; future researchers should explicitly explore this issue across a variety of spatial and temporal scenarios. In general, there are probably many important insights about range limits to be gained if researchers consider the effects of temporal environmental variation in more complicated landscapes.

## 4. Discussion

Temporal variation should have many different effects upon range limits, sometimes reducing ranges, and in others expanding them. This heterogeneity in outcomes can reflect both ecological and evolutionary reasons and the causal pathway through which variation exerts effects, and which traits and processes experience variation. A general insight into Darwinian evolution is that organisms adapt to their environments, including temporal variation. However, adaptations can become dependencies. Were temporal fluctuations cut off, some species would face extinction or shrunken ranges. The average annual temperature of Nome, Alaska, is below freezing. With constant conditions, water would always be frozen, ergo, little life. Because of seasonal pulses of warmth, however, a rich community of organisms with adaptations such as migration, hibernation, diapause and resource storage [31] for survival through harsh seasons, occupies the tundra, exploiting those pulses. Organisms have life history and physiological adaptations to exploit transient periods of benign conditions and survive stressful periods; some species require such variability to persist. Temporal variation for such species certainly permits larger ranges than otherwise expected. If species ‘eat variance’ there are consequences for the spatial ranges they can occupy.

A pervasive feature of living systems is that organisms have nonlinear relationships to environmental factors. Geometrically, nonlinearity means curvature. The model of exploitative competition equations (2.5) and (2.6) shows how temporal variation leads to systematic shifts in range limits, the qualitative direction of which can be discerned using Jensen's inequality. Often, intrinsic growth rate as a function of temperature is a strongly asymmetric unimodal function, increasing in exponential fashion at low temperatures, but plunging rapidly at high temperature [72,73]: in other words, concave up at lower temperatures, but concave downwards at higher temperatures. Variability in temperature might increase range limits on the low end, but shrink them on the high end, of a thermal gradient [72,73].

Another consequence of nonlinearity is that it can generate alternative stable states. The source–sink models above illustrate how moderate amounts of temporal variation can generate shifts between alternative states reflecting both ecological and evolutionary causes, transforming sink populations into persistent populations. Future work should examine patterns in temporal variability in both local growth rates and dispersal in more complex landscapes, along gradients, and with a wider array of dispersal patterns and genetic architectures. We conjecture that moderate temporal variability can foster an eco-evolutionary ratchet permitting a species to expand further along a continuous gradient, or within a patchwork of heterogeneous patches, than observed in otherwise similar, but constant, environments.

The positive effects of temporal variation on species' ranges might pertain to just a fraction of a species pool. Two well-known macroecological patterns are (i) range size is positively correlated with average local abundance, and (ii) most species are rare [4]. If species are rare within small ranges, temporal fluctuations aggravate local extinction risks and Allee effects simply compound those risks. The maximal size of dispersal pulses emanating from source populations will be constrained, given sparse source populations. Species typically rare or range-restricted are probably further constrained in their ranges by temporal environmental variation.

Another avenue for temporal variation to influence species’ range limits is via the evolution of phenotypic plasticity (e.g. [74]). Plasticity can extend the range of environments allowing population persistence and thereby facilitate range expansion—another indirect evolutionary consequence of temporal variation. Erikkson & Rafajlović explore this interplay between temporal variation and the evolution of plasticity at range margins [75].

In conclusion, temporal variability can surely shrink species ranges, but there are also clear circumstances when it might allow species to occupy larger ranges than expected in constant environments, for both ecological and evolutionary reasons. Grappling with these issues empirically will be challenging since it requires more information about population dynamics and microevolutionary processes than one usually has. Carefully sculpted microcosm studies might be a place to start [76]. Temporal variation is a ubiquitous feature of the natural world, currently altering owing to anthropogenic drivers such as habitat fragmentation and climate change. Working out the eco-evolutionary consequences of such variation for shifting species ranges is an important, looming challenge. There are significant conservation and management implications of our synthesis. There is a huge literature projecting future species' ranges, based on correlations between current climate and their distributions. Given that climate variability will change as well as mean conditions, it is important to recognize that projections need to take into account the diverse ways variation can influence range limits, sometimes constraining them, and sometimes permitting expansion. One potential management tool is deliberate introductions of species (e.g. moving species to a new range, or augmenting species in biocontrol efforts [77]). Ascertaining when temporal variability in immigration facilitates persistence in a novel environment for ecological and evolutionary reasons can help inform these efforts. For instance, theory suggests that translocation efforts might be enhanced given temporal variation in the number of individuals introduced, facilitating local adaptation [60,61,78]. This is just one example of the many potential ways that theory about temporal variation and range limits could inform conservation policy.

## Data accessibility

This article has no additional data.
